# The association between metamemory, subjective memory complaints, mood, and well-being: the Hungarian validation of Multifactorial Memory Questionnaire

**DOI:** 10.1186/s41235-023-00469-y

**Published:** 2023-02-14

**Authors:** Eszter Csábi, Emese Hallgató, Márta Volosin

**Affiliations:** 1 Present Address: Institute of Psychology, University of Szeged, Egyetem Utca 2, Szeged, 6722 Hungary; 2 Institute of Cognitive Neuroscience and Psychology, Research Centre for Natural Sciences, Magyar Tudósok Körútja 2, Budapest, 1117 Hungary

**Keywords:** Subjective memory complaints, Metamemory, Negative affect, Well-being, Memory strategies

## Abstract

The current study addressed the relationship between subjective memory complaints and negative affect, well-being, and demographic variables by investigating the Hungarian version of Multifactorial Memory Questionnaire. The original factor structure showed a poor fit on our data; therefore, principal component analysis was conducted on data from 577 participants, ranging in age from 18 to 92 years. Our analysis provided a six-component solution: *Satisfaction*, *Retrospective memory mistakes*, *Prospective memory mistakes*, *External Strategies*, *Internal Strategies,* and *Frustration*. To improve the reliability and internal consistency indicators we created four subscales by combining *Frustration* with *Satisfaction,* and *Retrospective* and *Prospective memory mistakes subscales*. Thus, we were able to preserve the factor structure similar to the original. Subjective memory complaints were correlated positively with anxiety and depression and were associated negatively with well-being. We found a slight positive correlation between age and memory ability, and age was associated negatively with the frequency of external strategy use. Individuals with higher education were satisfied with their memory, used more frequent external strategies. Furthermore, men were more satisfied with their memory and reported better memory ability, while women tended to use more external and internal strategies. Women also showed a higher level of anxiety and depression than men. In conclusion, self-reported memory changes are of particular importance because of their association with perceived mental health status and implications for later disease development.

**Supplementary Information:**

The online version contains supplementary material available at 10.1186/s41235-023-00469-y.

## Introduction

Metamemory is usually defined as knowledge about one’s own memory functioning including monitoring and control processes that enable the subjects to regulate their memory activity and content (Pannu & Kazniak, 2005; Shimamura, 2000; Simon et al., 2016). This knowledge encompasses several aspects such as factual knowledge about tasks, memory strategies, and the subject’s beliefs about their memory abilities (Pannu & Kaszniak, 2005). These ingredients are essential in directing memory processes in overall decision-making (Pannu & Kaszniak, 2005; Szajer & Murphy, 2013). According to imaging studies, the frontal and temporal lobes are involved in memory functioning and metamemory judgments (Pannu & Kaszniak, 2005; Szajer & Murphy, 2013).

Metamemory research has been considered relevant since previous studies revealed that subjective memory complaints can be related to neurological disorders (e. g. Korsakoff’s Syndrome, amnesia, frontal lobe damage, sclerosis multiplex, temporal lobe epilepsy) (Pannu & Kaszniak, 2005) and other diseases as well (e. g. preeclampsia, chronic fatigue syndrome, chronic pain) (Baecke et al., 2009; van der Werf & Vos, 2011). Age, gender, and the level of education have been commonly examined in relation to subjective memory complaints. According to the literature, the incidence of subjective memory complaints is more frequent in females than males (Jonker et al., 2000; Tomita et. al., 2014; van der Werf & Vos, 2011). Fritsch et al. (2014) suggest that frequent memory worries might cause their general worry about health concern (Fritsch et al., 2014). Furthermore, a higher prevalence of depression and negative affect among women compared to men is frequently associated with memory complaints (Gagnon et al., 1994; Jonker et al., 2000; Tomita et al., 2014). Self-reported changes in memory functioning also commonly appear in older adults (Joao et al., 2016; Minett et al., 2008; Park et al., 2007; Reid & MacLullich, 2006). The prevalence of subjective memory complaints among them is between 25 and 50% (Jonker et al., 2000) and the frequency of memory complaints shows a negative association with the level of education (Montejo et al., 2012). The most frequent problems in older adults include difficulty recalling names, misplacing household items, tip-of-the-tongue errors, and forgetting intentions (Burmester et al., 2015; Ossher et al., 2013). Together with objective cognitive decline measured by neuropsychological tests, the occurrence of subjective memory complaints is one of the DSM-5 Diagnostic Criteria for Mild Cognitive Impairment (MCI) (Ito et al., 2010). Recent studies revealed that older adults with subjective memory complaints have a high risk of developing dementia even in the absence of objective neuropsychological dysfunctions (intact performance on memory tasks) (Jessen et al., 2014; Mitchell et al., 2014; Rabin et al., 2017; Shaikh et al., 2021).

Other factors like depression and anxiety can contribute to metamemory functions. Complaints of declining memory are often reported among people with depressive and anxious symptoms (Balash et al., 2010; Balash, 2013; Reid & MacLullich, 2006; Minett et al., 2008; Yates et al., 2017). A study by Rowell et al. (2015) demonstrated that memory complaints and objective memory were significantly correlated for all age groups. Furthermore, higher negative affect was also associated with more memory complaints, irrespective of age. Moreover, Minett et al. (2008) found that subjective memory complaints were associated with depressive symptoms rather than objective cognitive performance. The presence of depressive symptoms because of negative effects and mood is related to well-being. Several studies revealed a relationship between memory complaints and diminished well-being (Mol et al., 2007; Waldorff et al., 2008). Maki et al. (2014) found that memory complaints had a negative impact on self-rated quality of life in a group of MCI patients compared to the non-clinical group. Thus, forgetfulness can be a negative predictor of the quality of life or it can be a bidirectional link between self-reported memory changes and quality of life. The association between memory complaints and reduced well-being might be mediated by depression (Montejo et al., 2012; Verhaeghen et al., 2000). Another possible explanation for the association between forgetfulness and a low level of quality of life may be the fear of dementia. Many people are afraid that changes in memory functions and forgetfulness are indicating early dementia (Commissaris et al., 1994).

To assess subjective memory complaints and metamemory functions Troyer and Rich (2002) designed the Multifactorial Memory Questionnaire (MMQ) that measures different aspects of memory including satisfaction with one’s memory (*Satisfaction*), perception of everyday memory ability (*Ability*), and use everyday memory strategies and aids (*Strategy*) (Troyer & Rich, 2002). The strengths of the questionnaire are that it is short, multi-dimensional, and easy to administrate. MMQ tends to focus on problems with recent memory (e. g. remembering names) and on strategies relevant to everyday life (e.g., repetition, written aids) (Troyer & Rich, 2002; Troyer et al., 2019). Other studies demonstrated only a weak (Crumly et al., 2014; Rabbitt & Abson, 1990) or no correlation between MMQ and objective memory performance (Jungwirth et al., 2004; Simon et al., 2016).

Taking these findings into consideration, the aim of the present study was to investigate the Hungarian version of MMQ and to explore its psychometric properties, thus providing a self-report questionnaire to identify subjective memory complaints. First, we tested the Hungarian translation of MMQ regarding means, internal consistency, construct validity, and test–retest stability. Second, we explored the underlying component structure by confirmatory factor analysis (CFA) followed by principal component analysis (PCA). Third, we examined how memory complaints are associated with demographic variables (such as age, gender, and education), depressive and anxious symptoms, and subjective well-being. We hypothesized that higher scores on MMQ—*Satisfaction* and MMQ—*Ability* are associated positively with education and well-being and associated negatively with age and the level of depression and anxiety. Moreover, we assumed that the frequency of strategy use has a negative association with well-being and a positive association with age and the level of depression and anxiety.

## Methods

### Participants

Participants over the age of 18 years were recruited via email and social media. They were asked to fill out an online questionnaire. A small sample of the participants completed the tests twice, the second occasion being within 1–3 weeks after the first completion. The purpose of the repeated completions was to screen for the test–retest reliability and items to which responses were not stable over time (e.g., it is difficult to judge the correct answer or the variable in question changes over a time span of up to 1–3 weeks). Respondents were given a unique identification number, which was used to match the responses belonging to the same person. Some participants older than 60 years filled out the questionnaire on paper, and their responses were coded identically to those participating in the online study.

#### Repeated data collection

A total of 474 completions were received. Of these, 68 identifiers were only used once, meaning that they did not actually complete the questionnaire more than once. There were 177 identifiers that were found twice as expected. In addition, there were 15 identifiers with which three completions were received (typically there was another completion immediately on the day of the first completion, followed by another one 1–3 weeks later). In these cases, the first of the completions received on the same day was taken into account. In addition, there were 3 identifiers with which 4 completions were received. In two of the latter cases, when demographic characteristics were taken into account, it became clear that in fact, two different persons (using the same identifier) had completed the questionnaires twice, according to the instruction—in their case, their identifier numbers were subsequently distinguished. In the remaining case, where 4 completions were received with the same identifier, it was found that 2 completions belonged together, taking into account the demographic data, and two others showed no identity with each other or with other data and were excluded from the analysis.

In order to check the reliability of the data, for the 195 (duplicate) completers identified in this way, we also examined whether their demographic data were meaningful and whether they were identical (or nearly identical) across the two completions. We excluded all respondents who gave a non-numerical answer to the question on how many years of schooling they had attended in their lifetime (e.g., they wrote “a lot”), and also those whose age or education (in years) differed by more than 1 year between the two completions, and who gave a different type of residence (e.g., village, town) or gender between the two completions. Given that differences between individuals were not important in the study of this question, health status was not considered as an exclusion criterion.

As a result, we conducted our analysis with data from a total of 157 participants (mean age *M* = 33.83 (SD = 12.53) years, minimum 18 to maximum 70 years; mean education *M* = 15.96 (SD = 3.75), minimum 8 to maximum 40; 51 men, 106 women) two-time respondents. The median number of days between the two competitions was 11 days, *M* = 11.32, SD = 3.26. A summary of the descriptive statistics of single and repeated data collection is illustrated in Table 1.

**Table 1:** Summary of the descriptive statistics of the repeated and single data collections

		Mean	Median	SD	Minimum	Maximum
Repeated data collection (*N* = 157)	Age	33.8	30	13.7	18	70
	Education	15.9	15	3.7	8	40
Single data collection (*N* = 577)	Age	38.2	34	16.1	18	92
	Education	16.2	16	3.6	5	40

#### Single or first data collection

The final sample consisted of 577 participants (mean age *M* = 38.2 (SD = 16.1) years (minimum 18 to maximum 92 years); mean education *M* = 16.2 (SD = 3.6), minimum 5 to maximum 40; 162 men and 414 women). A total of 577 completions were utilized consisting of single completions (*n* = 317) and the first completion of the repeated data collection irrespectively of the presence or meaningfulness of second completions (*n* = 260). A summary of the descriptive statistics of samples participated in single and repeated data collection is illustrated in Table 1.

Before completing the questionnaires, all participants provided informed consent. The study was conducted in accordance with the Declaration of Helsinki and the protocol was approved by the United Ethical Review Committee for Research in Psychology, Hungary (EPKEB; Reference number: 2021-07).

### Tasks

At the beginning of the questionnaires, we asked about the demographic data (e.g., gender, age, level of education, type of settlement, health condition), followed by the psychological questionnaires detailed below. The questionnaires took 20–25 min to complete. It is important to note that this study was conducted as a part of a project in which not only the Hungarian version of the MMQ but also of the Cognitive Failure Questionnaire (CFQ—Broadbent et al., 1982) was aimed to be validated, so the CFQ was included into questionnaire battery as well. However, as the focus of the present paper is to report the validation of MMQ, results on CFQ are reported elsewhere (Volosin et al., 2023).

#### Multifactorial Memory Questionnaire (MMQ)

MMQ is a self-report questionnaire designed to assess different aspects of self-reported memory across three scales. MMQ—*Satisfaction* consists of 18 items that measure one’s satisfaction with memory functioning. Higher scores on this subscale indicate higher satisfaction. The MMQ—*Ability* scale contains 20 items that assess the perception of everyday memory ability and the most common memory problems over the previous two weeks. Individuals with higher scores on this scale have a better subjective impression of their memory capabilities. The *Strategy* scale consists of 19 items that assess the frequency of strategy use in everyday life. It is important to note that this subscale indicates only the frequency of use and not the reason for using the aids. Thus, individuals who use more strategies could have good memory and satisfaction because they do use more strategies (Troyer & Rich, 2018). The original version of the test contains 57 items and the participants have to rate each item on a 5-point Likert scale (1 = never, 2 = rarely, 3 = sometimes, 4 = often, 5 = always). The MMQ—*Satisfaction* subscale consists of the following eleven reverse items: 2,4,5,7,8,10,11,14,15,16,18. Higher scores on MMQ subscales indicate higher satisfaction with memory functioning, better memory abilities and fewer memory problems, and greater use of strategies (Troyer & Rich, 2002, 2018). The original MMQ was validated in a sample of English-speaking, community-dwelling, middle-aged and older adults (Troyer & Rich, 2002; Troyer et al., 2019).

The MMQ was translated from English into Hungarian by the authors (ECS, MV). Then, the two translations were compared to produce a finalized version which was translated back into English by a Hungarian–English bilingual person. The resulting English version of MMQ was compared to the original English version, and their similarity was considered to be satisfactory. The Hungarian version of MMQ is provided at the following link: www.baycrest.org/mmq.

#### Cognitive Failure Questionnaire (CFQ)

CFQ is a widly utilized tool to measure the frequency of cognitive failures, for example, failing to remember someone’s name, or turning in the wrong direction on a familiar road (Broadbent et al., 1982). In the Hungarian version of CFQ (Volosin et al., 2023), the participants have to indicate in a 5-point Likert scale (from 0 to 4) the perceived frequency of cognitive lapses in the last 6 months. Higher points represent more perceived cognitive failures.

#### World Health Organization five well-being index (WHO-5)

WHO-5 is a 5-item rating scale used to measure current mental well-being. The participants have to rate the perceived frequency of each item in the past two weeks on a 4-point Likert scale (0 = at no time to 3 = all of the time). Higher scores represent higher well-being (Susánszky et al., 2006; WHO, 1998).

#### Patient Health Questionnaire (PHQ-9)

The PHQ-9 is a 9-item self-reported questionnaire designed to evaluate the presence of depressive symptoms during the prior 2 weeks. The participants have to answer on a 4-point Likert scale about the frequency of the occurrence of each symptom (0 = not at all to 3 = nearly every day) and have to rate the severity of the symptoms (caused difficulties in work, household, or social relations). Higher scores indicate a higher level of depressive symptoms (Kroenke et al., 2001).

#### Beck Anxiety Inventory (BAI)

BAI is a self-administrated questionnaire of the most common symptoms of anxiety. The Hungarian version of the test consists of 21 items; each item is rated on a 4-point Likert scale. The participants have to indicate how much they have been bothered by the symptoms during the past month (Beck et al., 1988; Perczel-Forintos et al., 2018).

### Statistical analysis

#### Identifying time-unstable items

A subgroup of participants was asked to fill out the questionnaires two times within 1–3 weeks. These test–retest methods allowed us the identification of items that appeared to be unstable between the two-time points of responding. All items and values which suggested instability are highlighted in bold, but we have not excluded these items from further analysis, and the final version of the Hungarian MMQ. The item analysis with the unstable items is presented in Additional file 1.

#### Investigating component structure and psychometric correlates

Confirmatory factor analysis (CFA) was conducted to evaluate the 3-factor (Troyer & Rich, 2002) and 4-factor models (Fort et al., 2004; Raimo et al., 2016); however, these models did not adequately fit the data (see Results). Therefore, we also conducted principal component analysis (PCA) using parallel analysis (PA) as the extraction method. At this point, is important to highlight that both exploratory factor analysis (EFA) and PCA could be a reasonable choice to further analyze our data as these techniques are related, and while EFA aims to identify latent constructs underlying the data, the goal of the PCA is to reduce the number of variables by summarizing into principal components explaining the maximum amount of total variance (for a review, see, e.g., Alavi et al., 2020). However, we chose PCA over EFA because of two reasons. First, we aimed to find the most adequate way to summarize the dimensions measured by MMQ components while explaining the maximum amount of information from the variables. The second reason was that previous studies (Fort et al., 2004; Raimo et al., 2016) also applied PCA, and using the same type of data reduction method would lead to better comparability with the literature. Parallel analysis (PA) was chosen instead of Kaiser’s rule (i.e., eigenvalue > 1) because in the latter case components are often over-extracted, which might lead to the misinterpretation of spurious components (Franklin et al., 1995). In contrast, PA generates a random data set beside the actual data, and only components with higher eigenvalues compared to those of the simulated data are considered to be significant (Horn, 1965). We utilized Oblimin rotation with the suppression of component loadings below 0.3.

After defining the component structure, Cronbach’s alpha was calculated on the data from single data collection, as well as test–retest reliability was measured by Pearson’s correlation coefficients between the summarized MMQ scores at the first and second completions (repeated data collection).

Finally, using data from the single data collection, we assessed the MMQ scores’ Pearson’s correlation with age, education, depressive and anxiety symptoms, well-being, and also possible gender differences. Statistical analyses were conducted in jamovi 2.2.5 (The jamovi Project, 2021).

## Results

### The component structure of the MMQ

Confirmatory factor analysis was applied to test the fit of both three-factor and four-factor models to assess the best-fitting structure. The 3-factor model (*Satisfaction Ability*, *Strategy*) was based on the originally proposed MMQ structure by Troyer and Rich (2002). The 4-factor model was originating from those studies that revealed that the *Strategy* scale is divided into two subscales (*External* and *Internal strategy*) (Fort et al., 2004; Raimo et al., 2016; Troyer et al., 2019). Table 2 summarizes the fit statistics for both models. The sample size in the present study (*N* = 577) can be considered to result in an adequate statistical power for factor analysis (see Kyriazos, 2018).

**Table 2:** Summary of 3-factor model and 4-factor model

	CFI	TLI	RMSEA	SRMR	*X*^2^ (df)
3-Factor model Troyer and Rich (2002)	0.780	0.771	0.062	0.065	4904.940 (1536)
4-Factor model Fort et al., (2004)	0.804	0.796	0.059	0.065	4445.549 (1478)
4-Factor model Raimo et al. (2016)	0.791	0.783	0.060	0.065	4724.648 (1533)

*CFI* Comparative fit index, *TLI* Tucker–Lewis index, *RMSEA* Root mean square error of approximation, *df* degrees of freedom. All *X*^2^ tests were significant at *p* < 0.001

These models did not adequately fit the data as CFI and TLI > 0.95, RMSEA < 0.06 and SRMR < 0.08 indicate a good fit (Chen, 2007; Hu & Bentler, 1999); therefore, we conducted PCA parallel analysis along with Oblimin rotation, suppressing component loadings below 0.3. Bartlett’s test of sphericity was significant (*Χ*^2^(1596) = 16,274.131, *p* < 0.001) and the overall Kaiser–Meyer–Olkin (KMO) measure of sample adequacy value was 0.944 (item values between 0.785 and 0.972), suggesting that the data were adequate for PCA. Parallel analysis revealed that the items loaded to six components (see Table 3).

**Table 3:** Component loadings and uniqueness values of MMQ items when utilizing PCA to extract components

MMQ Items	Component						Uniqueness
	1	2	3	4	5	6	
SA07	0.866						0.209
SA12	− 0.825						0.307
SA05	0.823						0.318
SA17	− 0.815						0.309
SA18	0.805						0.259
SA10	0.786						0.274
SA01	− 0.760						0.317
SA15	0.758						0.352
SA08	0.755						0.370
SA06	− 0.745						0.378
SA02	0.719						0.359
SA11	0.688						0.381
SA04	0.586						0.530
SA03	− 0.514						0.626
A02		0.755					0.443
A01		0.728					0.520
A05		0.647					0.406
A06		0.615					0.501
A13		0.520					0.484
A08		0.488	0.337				0.403
A18		0.481	0.343				0.449
A11		0.460					0.665
**ST02**		**0.388**					**0.662**
A15		0.367					0.551
A14			0.662				0.365
A09			0.629				0.472
A20			0.498				0.436
A07			0.487				0.441
A10	0.308		0.460				0.553
A17			0.430				0.596
A19		0.365	0.421				0.511
**ST09**			**0.383**				**0.744**
A12	0.317		0.370				0.612
A03			0.313				0.575
A16			0.308				0.628
A04			0.302				0.679
ST10				0.801			0.380
ST05				0.780			0.411
ST01				0.654			0.537
ST18				0.595			0.582
ST15				0.566			0.566
ST12				0.509			0.545
ST07				0.422			0.718
ST14					0.635		0.569
ST13					0.632		0.528
ST04					0.605		0.621
ST03					0.590		0.616
ST11					0.556		0.599
ST06					0.522		0.697
ST17					0.428		0.598
ST08			0.308		0.422		0.622
ST19					0.373		0.657
ST16					0.350		0.806
SA13						− 0.678	0.424
SA16						0.584	0.421
SA09						− 0.464	0.698
SA14						0.421	0.496

*SA = Satisfaction* subscale; *A = Ability* subscale; *ST = Strategy* subscale. 1 = *Satisfaction*; 2 = *Prospective memory mistakes*; 3 = *Retrospective memory mistakes*; 4 = *External Strategy*; 5 = *Internal Strategy*; 6 = *Frustration*

We marked with bold those items that loaded different scale in our analysis than in the original version of MMQ

The first component explained 16.864% of the variance (Sum of Squared loading of 9.612) and included 14 items and item 2 from the *Strategy* subscale evaluating overall contentment with one’s memory ability (*Satisfaction*). The second component accounted for 8.45% of the variance (Sum of Squared loading of 4.819) and included 10 items evaluating the perception of everyday memory ability and general memory mistakes (*Retrospective memory mistakes)*. The third component explained 8.105% of the variance (Sum of Squared loading of 4.620) and included 12 items and item 9 from the *Strategy* subscale assessing also the perception of everyday memory, especially prospective memory mistakes (*Prospective memory mistakes)*. The fourth component accounted for 6.336% of the variance (Sum of Squared loading of 3.661) and included 7 items assessing the use of every memory external strategy and aid (*External Strategy*). The fifth component accounted for 6.115% (Sum of Squared loading of 3.486) and included 10 items evaluating mnemotechnics (*Internal Strategy*). The sixth component explained 3.639% of the variance (Sum of Squared loading of 2.074) and included 4 items assessing general worry about one’s memory functioning (*Frustration*). The six-component solution explained 49.513% of the variance. The uniqueness values of items ranged from 0.259 to 0.806. To characterize each participant with a single value, the sum of each MMQ subscale score was computed. The uniqueness values are presented in Table 3.

Although the parallel analysis revealed that the items loaded to six components, we combined the *Frustration* with *Satisfaction* subscale because the former contains four items characterized with low test–retest reliability (*r*(155) = 0.509, *p* < 0.001) and internal consistency (*α* = 0.70). Furthermore, due to the high overlap between the *Retrospective memory mistakes* and *Prospective memory mistakes subscales,* we also combined these two subscales into one and named *Ability* subscale like in the original questionnaire by Troyer and Rich (2002). Thus, we could better preserve the factor structure similar to the original than with the six-component model. Moreover, the subscales provide better reliability indicators.

There were two items, ST02 (“Ask someone to help you remember something or to remind you to do something”) and ST09 (“Use a routine to remember important things, like checking that you have your wallet and keys when you leave home”*)* that belong to the *Strategy* subscale in the original version of MMQ (Troyer & Rich, 2002), but our PCA analysis revealed that they loaded on *Ability* subscale. We calculated Cronbach’s alphas for the *Ability* subscale with (*α* = 0.911) and without these two items (*α* = 0.917) and the removal of the items only slightly improved the internal consistency of the subscale. Therefore, we did not remove these two items from the final version of the questionnaire, but we also did not include them in further analysis such as reliability, internal consistency, and the total score of *Ability* and *Strategy* subscales. We marked with bold these items in Table 3.

### Test–retest reliability

The test–retest reliability was assessed in a subgroup of 157 participants who completed the questionnaires in two sessions within 1–3 weeks. The median number of days between the two competitions was 11 days, M = 11.32, SD = 3.26. The test–retest reliability was high in *Satisfaction* (*r*(155) = 0.896, *p* < 0.001) and *Ability* (*r*(155) = 0.822, *p* < 0.001) subscales. *External Strategy* (*r*(155) = 0.769, *p* < 0.001) and *Internal Strategy* subscales (*r*(155) = 0.743, *p* < 0.001) showed adequate reliability (Strauss et al., 2006). The item analysis is presented in Additional file 1.

### Internal consistency of scores on the Hungarian version of MMQ

The internal consistencies of the scores on the items constituting each MMQ dimension were examined using Cronbach’s α coefficients. Based on Cicchetti’s (1994) recommendation, for *Satisfaction* (α = 0.93) and *Ability* (α = 0.91) α were excellent. For *External (*α = 0.77) and *Internal Strategy (α* = 0.75) α were fair*.*

#### Construct validity

As for convergent validity, we found a strong negative correlation between the total score of CFQ and *Ability* subscale (*r*(575) = − 0.832, *p* < 0.001). The original (Broadbent et al., 1982) and the Hungarian version of CFQ (Volosin et al. 2023), as well as further studies (e.g., Goodman et al., 2022; Merckelbach et al., 1996; Tirre, 2018) suggested that cognitive failures represent a single factor. Some other studies revealed 2–7 components or factors underlying CFQ scores (e. g. Bridger et al., 2013; Eser et al., 2020; Larson et al., 1997). Rast et al. (2009) demonstrated a three-component solution: Forgetfulness, Distractibility and False Triggering. To confirm convergent validity, we correlated *Ability* subscale of MMQ with the Forgetfulness subscale of CFQ and we found a strong negative correlation between the two scales (*r*(575) = − 0.819, *p* < 0.001). The Cronbach’s alpha of CFQ was 0.920.

### Psychometrical correlates

We found a significant positive correlation between the *Satisfaction* and *Ability* subscales indicating that individuals with better memory abilities are more satisfied with their memory. *External* and *Internal* Strategy subscales are correlated negatively with *Satisfaction* and *Ability* subscales suggesting that individuals who have more memory problems and less satisfied with their memory utilize more external and internal strategies. Furthermore, *External* and *Internal Strategy* subscales showed a positive correlation with each other. The results are presented in Table 4.

**Table 4:** Pearson's correlation coefficients between MMQ subscales, depression, anxiety, and well-being

	Satisfaction	Ability	External strategy	Internal strategy
*Satisfaction*				
Ability	0.660***			
External strategy	− 0.339***	− 0.435***		
Internal strategy	− 0.289***	− 0.422***	0.474***	
PHQ	− 0.438***	− 0.465***	0.185***	0.204***
BAI	− 0.348***	− 0.414***	0.304***	0.271***
WHO	0.306***	0.292***	− 0.032	− 0.056

***p < 0.001

The internal consistency of PHQ, BAI, and WHO was good in our dataset (Cronbach’s alpha of PHQ was 0.832; Cronbach’s alpha of BAI was 0.915; and Cronbach’s alpha of WHO was 0.807). *Satisfaction* and *Ability* subscales demonstrated significant negative correlations with anxiety and depressive symptoms, suggesting that individuals who described themselves as more depressive or anxious reported more memory complaints and less satisfied with their memory abilities. Moreover, individuals with a higher level of affective symptoms used memory aids and strategies more often. Well-being showed a significant positive correlation with *Satisfaction* and *Ability* subscales, while there were no correlations between well-being and strategy subscales. The correlations are presented in Table 4.

### The relationship between MMQ subscales age and education

Age showed a weak, significant positive correlation with *Ability* subscale (*r*(575) = 0.099, *p* = 0.018) and a significant negative correlation with *External Strategy* (*r*(575) = − 0.200, *p* < 0.001) and *Internal Strategy* subscales *(r*(575) =  − 0.106, *p* = 0.011). There was no correlation between age and *Satisfaction* subscale (*r*(575) =  − 0.026 *p* = 0.541).

We found a weak positive correlation between education and *Satisfaction* (*r*(575) = 0.107, *p* = 0.010) and *External Strategy* (*r*(575) = 0.152, *p* < 0.001) subscales. There was no relationship between education and the other MMQ subscales: *Ability r*(575) = 0.049, *p* = 0.241; *Internal Strategy*: *r*(575) = 0.033, *p* = 0.428).

### Partial correlation

As aging is often associated with an enhanced level of depression and anxiety, we conducted partial correlations to reveal a more straightforward relationship between these variables and MMQ scores. When the effect of aging and education was controlled, the significant relationship between well-being, anxiety, depression, and MMQ subscales remained. The correlations are presented in Table 5.

**Table 5:** Pearson's correlation coefficients between MMQ scales and affective variables after controlling and age and education

	Satisfaction	Ability	External strategy	Internal strategy
PHQ	− 0.442***	− 0.454***	0.178***	0.195***
BAI	− 0.366***	− 0.403***	0.274***	0.255***
WHO	0.300***	0.292***	− 0.048	− 0.061

***p < 0.001

When we controlled depression and anxiety level, there remained a weak correlation between age and *Satisfaction* (*r*(575) = -0.117, *p* = 0.006) and *External Strategy* (*r*(575) = − 0.127, *p* = 0.003). Based on these data we suggest that MMQ scores might reflect a general worry about cognitive decline. The relationship between education and *External Strategy* subscales was still observed (*r*(575) = 0.147, *p* < 0.001).

### Gender differences in MMQ subscales, depression, and anxiety

Due to the skewed distribution of MMQ scores (*Satisfaction*: men: Shapiro–Wilk’s *W* = 0.871, *p* < 0.001; women: *W* = 0.908, *p* < 0.001; *Ability*: men: Shapiro–Wilk’s *W* = 0.908, *p* < 0.001; women: *W* = 0.945, *p* < 0.001; *External Strategy*: men: Shapiro–Wilk’s *W* = 0.978, *p* = 0.012; women: *W* = 0.988, *p* = 0.002; *Internal Strategy*: men: Shapiro–Wilk’s *W* = 0.863, *p* < 0.001; women: *W* = 0.941, *p* < 0.001), we evaluated gender differences using the Mann–Whitney test.

We revealed significant differences between the genders on *Satisfaction* (*U* = 29,202, *p* = 0.016; Med_women_ = 57 vs. Med_men_ = 60), *External* (*U* = 25,358, *p* < 0.001; Med_women_ = 11 vs. Med_men_ = 14) and *Internal Strategy* subscales (*U* = 29,965, *p* = 0.046; Med_women_ = 6 vs. Med_men_ = 5). Moreover, we revealed a trend in the *Ability* subscale (*U* = 300,021, *p* = 0.05; Med_women_ = 62 vs. Med_men_ = 64.5) between the genders. These results indicate that men are more satisfied with their memory and tended to report better memory abilities than women, and women utilize external and internal memory strategies and aid more often than men.

We also revealed significant differences in depressive symptoms and the level of anxiety; women demonstrated more depressive symptoms (*U* = 28,465, *p* = 0.005; Med_women_ = 9 vs. Med_men_ = 7) and a higher level of anxiety than men (*U* = 26,864, *p* < 0.001; Med_women_ = 28 vs. Med_men_ = 26). We did not find differences in well-being between genders (*U* = 29,868, *p* = 0.256; Med_women_ = 8 vs. Med_men_ = 8).

## Discussion

The aim of the present study was to investigate the psychometric characteristics of the Hungarian version of MMQ and the relationship with demographic variables, negative mood, and well-being. Our analysis revealed a six-component model. To improve the reliability and internal consistency indicators we combined *Frustration* and *Satisfaction* subscales and *Retrospective* and *Prospective memory mistakes* subscales. Thus, we were able to preserve the factor structure similar to the original and provide better reliability indicators. We found a correlation between the MMQ subscales indicating that individuals with better memory abilities were more satisfied with their memory, and tended to use external and internal strategies less frequently. Age showed a slight positive association with memory ability and a negative association with the usage of external and internal strategies. A weak positive correlation was found between education and satisfaction and the frequency of external strategy use indicating that individuals with higher education are more satisfied with their memory and use more frequent external strategies. We observed that men were more satisfied with their memory and tended to report better memory abilities, and women utilized external and internal memory strategies and aid more frequently than men. Finally, we detected that anxiety and depression, and well-being influence on memory ability and metamemory judgments. Individuals with a higher level of depression and anxiety reported more memory complaints, they were less satisfied with their memory abilities and used memory aids and strategies more often.

Our finding demonstrated that the items loaded to six components accounting for 49.513% of the common variance in the data set. The first component corresponded to the factor “*Satisfaction*” similar to the original validation study of the MMQ (Troyer & Rich, 2002). The second and third components corresponded to the components “*Retrospective memory mistakes*” and “*Prospective memory mistakes.”* In previous studies, these two subscales were included within one factor (“*Ability*”) (Fort et al., 2004; Raimo et al., 2016; Shaikh et al., 2021; Simon et al., 2016; Troyer & Rich, 2002 van der Werf & Vos, 2011). Due to the high overlap between *Retrospective memory mistakes* and *Prospective memory mistakes* subscales we combined these two scales into one and named *Ability* subscale like in the original questionnaire by Troyer and Rich (2002). The fourth and fifth components correspond to the “*External*” and “*Internal Strategies*,” whereas they were included within one factor in the original study (Troyer & Rich, 2002), and the Brazilian version of MMQ (Simon et al., 2016). Our results are in line with the French (Fort et al., 2004) and Italian versions (Raimo et al., 2016) of MMQ. These studies also proposed subdividing the factor “*Strategy*” scale into two separate factors, “*Internal Strategies*” and “*External Strategies*.” External strategies can reinforce new learning and remembering something by utilizing external tools (e. g., using the calendar or a timer or alarm to remember something, writing down things to do, etc.). Internal strategies are those mental strategies that help the subjects optimize their memory functions (e. g. creating a mental image, organizing the information, making an acronym), and may require more cognitive effort than external strategies (Fish et al., 2008; Fort et al., 2004). Separating external and internal strategies subscales can be useful in assessing the use of different kinds of strategies in various diseases or in different ages and planning cognitive interventions. The differences between the factor structures can be explained by translational aspects and cultural differences that might affect the perception of memory ability. Furthermore, methodological differences can also contribute to the variation of component structure, such as the type of analyses (principal component analysis versus confirmatory factor analysis), sample size (ranging from 87 to 1075), and the characteristics of the samples with regard to age, education, and health status (Shaikh et al., 2021; Troyer et al., 2019). The sixth component corresponded to the “*Frustration”* subscale contains 4 items and assesses the level of frustration/anxiety about memory functioning. Due to the low reliability and internal consistency caused by the low item number, we combined the *Frustration* with *Satisfaction* subscale that led to an improvement in reliability indicators. By combining the subscales, the final version of MMQ contains four subscales that provide better reliability and internal consistency indicators and the structure of the questionnaire are more similar to the original version of MMQ.

Age showed a slight positive association with memory ability and a negative association with the usage of external strategy. In line with the observation of Szajer et al. (2013), these results suggest that individuals with older age might be more confident in their memory abilities; therefore, they use fewer external strategies. Consistent with these findings, when we controlled other factors such as anxiety, depression, and well-being, a positive correlation appeared between age and the *Satisfaction* subscale indicating that older age is related higher level of satisfaction with memory abilities. This relationship can explain the negative association between age and *External strategy* use that still remained after we controlled affective variables. These results correspond to findings from studies suggesting that older adults have more experience with time management, knowledge of their memory’s fallibility, and fewer distractions due to the cognitively less demanding retirement-related lifestyle. These factors provide them better opportunity to plan how they will remember to execute their tasks (de Winter et al., 2015; Garden et al., 2001; Henry et al., 2004). Taking all these results into consideration, Simon et al. (2016) suggest that in older age beliefs on one’s own memory efficacy could be weaker than in younger age. In contrast to our results, the original validation study of MMQ by Troyer and Rich (2002) failed to find a relationship between age and the MMQ subscales. The authors suggested that a significant association between age and MMQ would have emerged if younger adults were included in their sample (average age was 71.7 (SD = 9.9), range 39–91). Moreover, contrary to our results, previous validation studies of MMQ revealed a negative association between age and MMQ subscales suggesting that with advancing age, individuals tend to report more memory complaints, they less satisfied with their memory abilities and used external and internal strategies more frequently (Raimo et al., 2016; van der Werf & Vos, 2011) irrespective of screening of neurodegenerative disorders (Fort et al., 2004).

Our results are in line with those previous studies that revealed that a higher level of education is associated with a higher level of satisfaction and more frequent external strategy use (Fort et al., 2004; Raimo et al., 2016; van der Werf & Vos, 2011). Troyer and Rich (2002) demonstrated a significant positive association with MMQ—*Ability* and education. These results indicated that subjects with higher educational level were more satisfied with their memory ability and inclined to use external strategies more frequently than individuals with lower education level (Fort et al., 2004; Raimo et al., 2016; van der Werf & Vos, 2011). Comijs et al. (2002) suggested that individuals with higher education have more memory complaints as a consequence of diminished well-being indicating that they are more sensitive to the quality of cognitive performance. Bouazzaoui et al. (2010) demonstrated that older adults (over 61 years) preferentially used external strategies to adapt and compensate for memory decline because they required less cognitive effort than internal strategies (Bouazzaoui et al., 2010; Horhota et al., 2012). These results confirm the reduced cognitive resource hypothesis of aging which supposes that older adults experience difficulty in effortful self-initiated processes (Anderson et al., 2000).

Our results revealed that men were more satisfied with their memory and tended to report better memory abilities, while women utilized external and internal memory strategies and aids more frequently than men. These results contradict the studies suggesting that being male is associated with higher rates of subjective memory complaints (Jorm et al., 2004). Our results are in line with other studies which supposed that the female gender is related to a higher prevalence of memory complaints (Jonker et al., 2000; van der Werf & Vos, 2011). The possible explanation for these findings is that women tend to have higher prevalence rates of anxiety and depression which are strongly associated with memory complaints (Comijs et al., 2002; Jorm et al., 2004; Rowell et al., 2015; Yates et al., 2017) and might cause more frequent use of external and internal strategies. Our results can confirm this hypothesis because we also revealed a higher level of anxiety and depression in women than men. Consistent with these findings, previous studies demonstrated that negative affect is associated with enhanced self-monitoring and awareness of errors (Carrigan & Burkus, 2016) and these personality traits might cause the greater reported frequency of external and internal strategies. Moreover, we cannot rule out the possibility that women are prone to multitasking (e. g. caring for children or family members, managing the household) (Chung et al., 2021) which also can explain the frequent use of different types of strategies. These results imply that further studies should investigate the background of gender differences in subjective memory complaints and the use of memory aids at multiple levels.

We demonstrated that metamemory judgments and subjective memory complaints were associated with anxiety and depression suggesting that individuals with fewer mood symptoms were more satisfied, reported better memory ability and used memory aids and strategies less often. Also, we found that well-being was related to a better subjective impression of one’s memory capabilities and higher satisfaction with one’s abilities. These correlations were still observed after controlling for age and education. Our results are in tune with the previous literature showing that negative mood and poor well-being are risk factors for memory complaints (Simon et al., 2016). Consistent with these findings, a recent study by Quek et al. (2021) found that those who had concerns about their memory arising from a general disposition to worry rather than those who acknowledge memory difficulties as a result of a general tendency to negatively evaluate their own abilities. The authors suggest that memory complaint then may simply be an expression of underlying anxiety (Quek et al., 2021). A longitudinal study by Comijs et al. (2002) followed over 2000 respondents with an age range between 55 and 85 years for 6 years and revealed that memory complaints were associated with physical health problems, depressive and anxiety symptoms, low feelings of mastery, low perceived self-efficacy and high neuroticism. They conclude that older people complain about their memory and do not show an actual cognitive decline, one should be aware that these complaints might reflect psycho-affective or health problems. Moreover, they also supposed that physical disease may contribute to a lower feeling of well-being and poor motivation resulting in lower performance on cognitive tasks and more memory complaints (Comijs et al., 2002). A community-based study by Jorm et al. (2004) also supported that physical health and mental health are the strongest predictors of memory complaints. The relation is probably bidirectional because subjective memory problems lead to a negative perception regarding their health status, thus, they can have a general conception that their health is not good enough (Jorm et al., 2004). These findings imply that subjective memory complaints are probably mediated by other variables than objective performance, such as mood, and physical and mental health.

In conclusion, our study demonstrated that the Hungarian version of the MMQ has good psychometric properties and is useful for assessing subjective memory complaints and can be relevant to help design cognitive interventions in older adults who have memory complaints but no memory aids in everyday life. We believe that MMQ can help us to get a more sophisticated picture of memory processing, which can be useful for adequate rehabilitation programs as well. Moreover, it is important to investigate the self-reported memory changes in memory functioning because they may affect daily living and perceived health status and may indicate the development of later disease.

## Supplementary Information

**Additional file 1**. Supplemental Analysis.

## Data Availability

The datasets used and/or analyzed during the current study are available from the corresponding author upon reasonable request.
