# Supplementary material for: The association between metamemory, subjective memory complaints, mood, and well-being: the Hungarian validation of Multifactorial Memory Questionnaire
Source: Cogn Res Princ Implic. 2023 Feb 14;8:15. doi: 10.1186/s41235-023-00469-y (PMC9928992; doi:10.1186/s41235-023-00469-y)
Supplement: Supplementary file 1 [file 41235_2023_469_MOESM1_ESM.odt]

Supplementary A

Identifying time-unstable items

A subgroup of participants (n = 157; mean age M = 33.83 (SD = 12.53) years, minimum 18 to maximum 70 years; mean education M = 15.96 (SD = 3.75), minimum 8 to maximum 40; 51 men, 106 women) asked to fill out the questionnaires two times within 1-3 weeks. The median number of days between the two competitions was 11 days, M = 11.32, SD = 3.26.  These test-retest methods allowed us the identification of items that appeared to be unstable between the two-time points of responding.

In the repeated-measures survey, each person answered each question twice, and the extent to which these answers are similar can be examined from several different perspectives. First of all, the first and second responses to each question should be correlated. Given the level of measurement of the variable (ordinal) and the tied ranks, we computed Kendall's tau for each item. A high correlation with a positive sign means that if a participant answered with a high value the first time, they were asked a question, they answered with a high value the second time as well, and vice versa.

However, correlation per se does not fully indicate that the two responses were similar across the two completions. For example, it is possible that everyone answers a question with a low value (e.g., 1 or 2) the first time being asked, but then answers with a higher value (3 or 4) on the second occasion. In this case, the correlation can be very high, even though the answers are obviously inconsistent between the two measurements.

To take this into account, we computed the typical absolute differences between the responses (asked first vs. asked for the second time). It does not matter whether someone indicates a lower value first and a higher value second, or vice versa, the point is that there is a difference between the two responses. It is worth looking at this difference in absolute value to see which questions had the most different answers in the two-time points.

Finally, although the above-mentioned absolute difference is an important indicator of instability in responses, it does not differentiate noise and systematic bias. The issue can be resolved by constructing an indicator that takes into account the sign of the difference as well (response at first measurement minus response at second measurement) in addition to the absolute value of the difference. If this indicator is around zero on average, but the average of the absolute differences is high, then we can talk about noise. This can occur when, for example, it is difficult to judge whether a phenomenon occurs "a lot" or "moderately often" – it may not be clear what is typical, against which the person should give the answer. For this reason, it is possible that participants reach different conclusions about the same behaviour, even though the frequency of the behaviour has not actually changed between the two-time points. The other possibility is that the mean of the differences is typically positive or typically negative (i.e., non-zero). In this case, we can speak of a systematic shift, whereby participants typically indicate lower or higher values at the second response than at the first response. This may occur if the frequency of the observed behaviour changes between the two measurement dates, or if the person becomes more aware of a behaviour because of being explicitly asked about it at the first measurement, and thus pays more attention to it in the following days/weeks resulting in changed perception/execution of a particular behaviour.

These values are of course not independent of each other. Noise (large mean absolute deviations but near-zero mean deviations) reduces the correlation coefficient, while bias does not necessarily, but bias is also associated with large absolute deviations. Henceforth, it was considered to be indicative of noise if the Kendall tau correlation coefficient was less than 0.5 and the mean of the absolute deviations were greater than or equal to 0.5, while the mean of the deviations was less than 0.2/greater than -0.2. On the other hand, it was considered to be indicative of bias if the mean of the deviations were greater or equal to 0.2/less than or equal to -0.2.

We had to come up with these thresholds ourselves because we couldn’t find any guidelines we could follow for this particular use. The interpretation of correlation coefficients varies between disciplines (Akoglu, 2018), and 0.7 indicates a strong correlation in psychology using Pearson’s or Spearman’s correlation. However, Kendall tau coefficients are usually smaller than Spearman’s coefficients calculated on the same datasets (Fredricks & Nelsen, 2007) so in this case, a smaller value would already indicate a strong relationship. Furthermore, in an ideal case, these items should show a perfect relationship, so even an otherwise strong correlation (if it is a lot less than perfect) could be of concern for us. This is why we chose 0.5 as our threshold – anything below that is clearly less than perfect, even if it’s still quite strong.

In an ideal case, the mean absolute differences between the pairs would be zero. We aimed to flag items where this was very obviously not the case. We thought that if the absolute differences had a mean of 0.5, that would mean that on average, half of the participants rated the items differently (with a difference of 1 score on the Likert scale) between the two occasions – and one-half of all participants is anything but negligible. Since some people rated the same items even more differently between the two occasions (i.e., having a difference of 2-4 scores on the Likert scale), setting this threshold did not guarantee that we would observe differences in the case of 50% of the participants, as bigger differences increase the mean more than smaller differences. However, those also indicate higher instability on the particular items, so we sticked to this rule. In our case that resulted in flagging those items that got different ratings on the two occasions by at least 40% of our participants.

Lastly, looking at the raw differences (rather than their absolute values), our threshold of 0.2 would indicate that – on average – every fifth participant would systematically score higher by a score of 1 on the second occasion than on the first occasion – and vice versa for the negative values. We figured that this would indicate a non-trivial shift even if we take a possibly large noise into consideration, e.g., for every two participants scoring lower than previously by a score of 1, there would be three participants scoring higher by 1. In this example, the two (out of five) participants scoring lower and another two participants scoring higher would be simply noise that cancels each other out; the fifth participant however introduced a shift in the positive direction, and mathematically speaking, this would manifest as a shift of 0.2. All items and values which suggested instability are highlighted in bold, but we not excluded these items from further analysis, and the final version of Hungarian MMQ. Statistics are shown in Table 1.

Table 1. Item statistics including Kendall’s tau, mean and standard deviation of the absolute difference between the first and second completions irrespectively of the direction of the difference, and mean and standard deviation of the difference between the first and second completions.

|  | Time-unstable of type | Kendall’s tau | Absolute difference (first - second) | Difference (first - second) |
| --- | --- | --- | --- | --- |
| Mean | SD | Mean | SD |
| MMQ-SA    Item 1    Item 2    Item 3    Item 4    Item 5    Item 6    Item 7    Item 8    Item 9    Item 10    Item 11    Item 12    Item 13    Item 14    Item 15    Item 16    Item 17    Item 18 MMQ - A    Item 1    Item 2    Item 3    Item 4    Item 5    Item 6    Item 7    Item 8    Item 9    Item 10    Item 11    Item 12    Item 13    Item 14    Item 15    Item 16    Item 17    Item 18    Item 19    Item 20 MMQ-ST    Item 1    Item 2    Item 3    Item 4    Item 5    Item 6    Item 7    Item 8    Item 9    Item 10    Item 11    Item 12    Item 13    Item 14    Item 15    Item 16    Item 17    Item 18    Item 19 | Noise Noise Bias Noise Noise Noise Noise Bias Bias Bias + Noise Bias + Noise Noise | 0.69 0.61 0.52 0.71 0.71 0.65 0.71 0.56 0.36 0.63 0.67 0.67 0.52 0.46 0.64 0.59 0.71 0.70 0.63 0.63 0.60 0.53 0.58 0.56 0.67 0.59 0.64 0.58 0.58 0.54 0.55 0.61 0.55 0.45 0.61 0.56 0.52 0.59 0.61 0.39 0.64 0.44 0.73 0.59 0.46 0.40 0.51 0.70 0.52 0.53 0.48 0.37 0.45 0.52 0.49 0.50 0.54 | 0.39 0.41 0.41 0.24 0.34 0.51 0.33 0.42 0.97 0.50 0.43 0.41 0.76 0.89 0.33 0.69 0.39 0.39 0.37 0.51 0.62 0.71 0.52 0.31 0.47 0.50 0.50 0.48 0.36 0.48 0.48 0.45 0.48 0.51 0.48 0.54 0.62 0.49 0.68 0.79 0.13 0.48 0.50 0.20 0.75 0.85 0.83 0.57 0.76 0.71 0.48 0.29 0.90 0.21 0.83 0.81 0.66 | 0.57 0.73 0.54 0.54 0.62 0.76 0.67 0.74 1.18 0.78 0.72 0.73 0.87 0.88 0.59 0.81 0.63 0.68 0.68 0.66 0.77 0.83 0.62 0.60 0.63 0.64 0.58 0.68 0.58 0.67 0.62 0.58 0.67 0.72 0.66 0.65 0.69 0.71 0.80 0.80 0.48 0.85 0.69 0.61 0.96 0.95 1.05 0.72 0.93 0.74 0.74 0.63 1.04 .054 0.85 0.98 0.85 | -0.03 0.01 0.03 0.01 -0.05 -0.03 -0.11 -0.01 -0.07 0.08 -0.01 -0.04 -0.03 0.13 0.01 0.06 0.07 0.02 0.00 -0.10 0.10 0.16 0.20 -0.08 0.06 -0.18 0.03 -0.02 -0.06 -0.06 0.10 -0.01 -0.13 0.03 -0.04 0.00 0.18 0.02 -0.16 0.10 -0.02 0.16 0.04 0.10 0.04 -0.05 0.32 0.16 0.25 0.13 0.11 0.08 0.25 -0.03 0.21 0.12 0.03 | 0.70 0.84 0.68 0.59 0.71 0.92 0.74 0.85 1.53 0.92 0.84 0.84 1.15 1.25 0.68 1.07 0.73 0.78 0.78 0.83 0.99 1.08 0.78 0.67 0.78 0.79 0.77 0.83 0.68 0.82 0.78 0.74 0.81 0.88 0.81 0.84 0.91 0.87 1.04 1.12 0.50 0.96 0.85 0.63 1.22 1.28 1.30 0.90 1.17 1.02 0.88 0.69 1.36 0.58 1.18 1.27 1.08 |

Note: Time-unstable indicator is labeled noise when low Kendall’s tau is accompanied by high absolute difference, and it is labeled bias when both the absolute difference and the difference are considered large. All values which suggested instability are highlighted in bold. MMQ SA = Satisfaction subscale, MMQ A = Ability subscale, MMQ ST = Strategy subscale.
